# Supplementary material for: Solidification of Earth’s mantle led inevitably to a basal magma ocean
Source: Nature. 2025 Mar 26;640(8057):114–9. doi: 10.1038/s41586-025-08701-z (PMC11964929; doi:10.1038/s41586-025-08701-z)
Supplement: Supplementary file 1 — This file contains additional information, including values of physical parameters used in our model, the mathematical formalism we use to handle phase change and chemical partitioning in our simulations, analytical predictions and scaling analysis, regime diagrams calculations, and benchmark tests [file 41586_2025_8701_MOESM1_ESM.pdf]

nature portfolio

https://doi.org/10.1038/s41586-025-08701-z

Supplementary information

Solidification of Earth's mantle led inevitably to a basal magma ocean

In the format provided by the authors and unedited

Supplementary Information: Solidification of Earth's mantle led inevitably to a basal magma ocean

Charles-Édouard Boukaré[1,2], James Badro[1†] and Henri Samuel[1†]

[1]Université Paris Cité, Institut de Physique du Globe de Paris, CNRS, 1 rue Jussieu, 75005 Paris, France.
[2]York University, Department of Physics and Astronomy, 4700 Keele Street, M3J 1P3 Toronto, Canada.

Contributing authors: boukare@ipgp.fr; samuel@ipgp.fr; badro@ipgp.fr;
†These authors contributed equally to this work.

We use the label MT to refer to equations introduced in the Methods section of the *Main Text*.

1 Phase change

We assume thermodynamic equilibrium between solid and liquid at every time step, everywhere in the model domain. Phase change is therefore not limited by reaction kinetics but only by phase diagrams (which describe the solid-liquid equilibrium) and energy conservation. Phase change is treated differently for major and trace elements. For major elements, phase change is constrained by a parameterized phase diagram (Fig. S2) and an energy conservation equation. For simplicity, we neglect the latent heat of phase change for trace elements. Trace elements are simply distributed among the phases according to their respective partitioning coefficients.

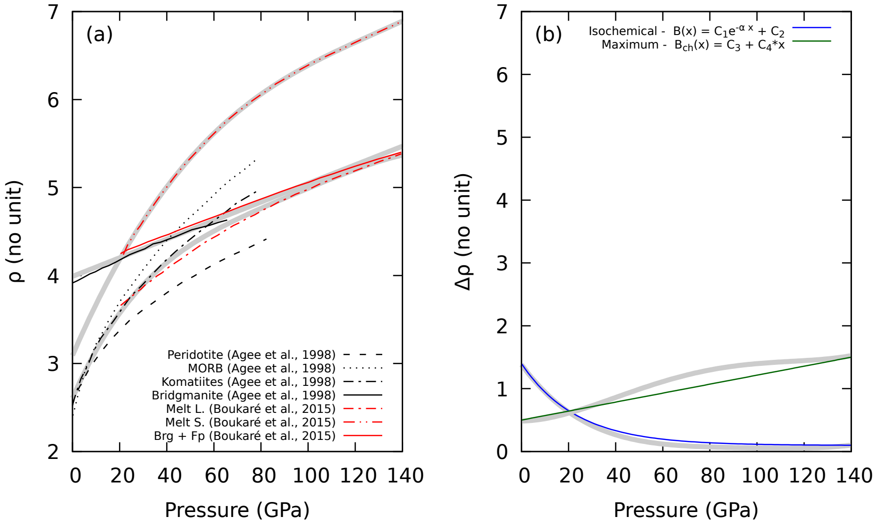

Fig. S1 Density contrast parameterization. (a) Density of solid and liquid silicate as function of pressure for various composition. (b) Density difference between solid and liquid. The thick grey lines correspond to the exact difference between the density curves shown on the left. The color lines depict the parameterization we used in this study. The iso-chemical density contrast is shown in blue ($\Delta\rho_1$ in MT Eq. (2)) and capture to the effect of highest compressibility of silicate melt compared to their solid couterparts. The chemical density contrast correspond to the density contrast between iron-rich and iron-free silicates ($\Delta\rho_2$ in MT Eq. (2)).

1.1 FeO partitioning - Equilibrium thermodynamics

We opted for an idealized binary loop to capture mantle melting. This simple approach allows us to capture the main influence of FeO partitioning on magma ocean evolution (Fig. S2):

- The first-order influence of FeO on both solid and liquid densities, which controls the gravitational stability of FeO-rich layers.
- The first-order role of FeO on the melting temperature, as FeO acts has an anti-freeze effect by shifting the melting curves towards higher values.
- FeO is slightly more compatible in the liquid phase than in the crystals (see binary loop in Fig. S2) .

After updating of the temperature field using MT Eq. (3), *i.e.* , prior to phase change, the dimensionless energy conservation in each computational cell (*i.e.*, volume control) of the numerical domain writes:

$$T_f - T_i = -(\Gamma_1 + \Gamma_2) S_t, \tag{1}$$

where T_f is the final temperature, T_i is the temperature before phase change (see MT Eq. (3)). Mass conservation of the melt fraction ($\phi = \phi_3 + \phi_4$) writes,

$$\phi_f - \phi_i = \Gamma, \tag{2}$$

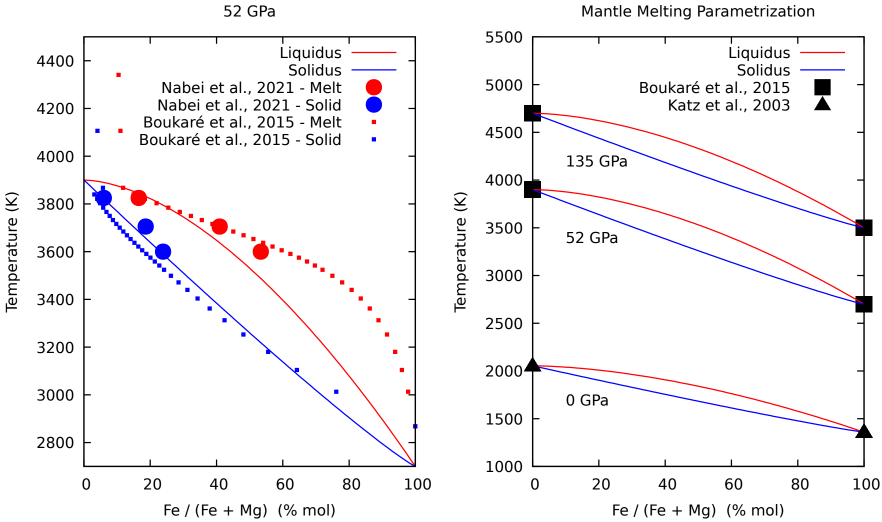

Fig. S2 Idealized binary loop model used to model solid-liquid melting relations in the Earth's mantle. This parameterization was obtained by fitting diamond anvil cell experiments [1] and self-consistent thermodynamic calculations [2]. This phase diagram has a primary role in the early Earth's mantle evolution. First, it controls the FeO chemical partitioning between solids and liquid, which has a first-order impact on the buoyancy of iron-rich silicates. Second, it controls the effect of FeO content on silicate melting temperature. This plays an important role on the formation of a basal magma ocean by remelting iron-rich solids at the CMB.

where ϕ_f is the final melt fraction, ϕ_i is the melt fraction before phase change, and $\Gamma = (\Gamma_1 + \Gamma_2)$. For each time step, after solving for the conservation of energy (*i.e.*, after one advection-diffusion time-step for temperature), the solid-liquid mixture is potentially out of equilibrium. We therefore consider three cases to treat this disequilibrium:

1) *The temperature is above the liquidus but the liquid fraction is lower than 1.* If the temperature is hot enough such that it remains above the liquidus after melting of the residual solids, melt fraction is set to 1. The melting temperature is updated using Eq. (1) according to the associated Γ.
2) *The temperature is below the solidus but the liquid fraction is larger than 0.* If the temperature is cold enough to remain below the solidus after the solidification of the residual melt, melt fraction is set to 0. The temperature is updated using Eq. (1) according to the associated Γ.
3) All other cases require a special treatment. The challenge is to determine Γ_1, Γ_2 and T_f such that T_f, the final melt fraction as well as the relative amount of FeO in the melt and the solid correspond to a thermodynamic equilibrium.

We describe below our approach to handle the case 3 mentioned above, which is a variant of the approach proposed in [3].

Springer Nature 2021 LaTeX template

Because the final temperature and the melt fraction must be at equilibrium, we have to solve the following system,

$$\begin{cases} T_i - \Gamma S_t = T_{eq} \\ \phi_i + \Gamma = \phi_{eq} \end{cases} \tag{3}$$

where T_{eq} is the equilibrium temperature between the solidus and the liquidus and ϕ_{eq} is the equilibrium melt fraction. Both quantities are constrained by the phase diagram. We need additional equations to close this set of equations *i.e.*, we need to solve the system (3) for only one unknown in addition to Γ).

The branches that describe the liquidus and solidus temperatures in the simplified phase diagram we use (Fig. S2) are parameterized as follows:

$$T_L = T_{\mathrm{FeO}} + (T_{\mathrm{MgO}} - T_{\mathrm{FeO}})\,(1 - x_l^m)\,, \tag{4}$$

$$T_S = T_{\mathrm{FeO}} + (T_{\mathrm{MgO}} - T_{\mathrm{FeO}})\,(1 - x_s)^n \tag{5}$$

where T_L is the liquidus temperature, T_S is the solidus temperature, T_{MgO} and T_{FeO} are the temperature of the pure end-members, x_l is the composition of the liquid in FeO, x_s is the composition of the solid in FeO, m and n are constant that we used to adjust the shape of the loop. At thermodynamic equilibrium we have:

$$T_L = T_S = T_{\mathrm{eq}}. \tag{6}$$

This yields an equation that relates the liquid and solid compositions:

$$x_l = \left[1 - (1 - x_s)^n\right]^{1/m}. \tag{7}$$

By the lever rule, we have,

$$\phi_{\mathrm{eq}} = 1 - \frac{x_l - x_{\mathrm{bulk}}}{x_l - x_s}, \tag{8}$$

where x_{bulk} is the bulk composition of the solid-liquid mixture in FeO. Using Equations (4), (7) and (8), we can rewrite the system of two equations (3) with only two unknown variables, Γ and x_s,

$$\begin{cases} T_i - \Gamma S_t = T_S(x_s) \\ \phi_i + \Gamma = \phi_{\mathrm{eq}}(x_l(x_s), x_s) \end{cases} \tag{9}$$

Due to the non-linearity of Eq. (4) and Eq. (7), we solve this set of equations iteratively by minimizing the following function for x_s and Γ using a Newton-Raphson method in two dimensions,

$$G(x_s, \Gamma) = \left[T_i - \Gamma S_t - T_S(x_s)\right]^2 + \left[\phi_i + \Gamma - \phi_{eq}(x_l(x_s), x_s)\right]^2 \tag{10}$$

Γ_1 and Γ_2 can be retrieved *a posteriori* when the final melt fraction, liquid and solid compositions are known.

While we focused on the limiting case where liquid and solid phases remain at thermodynamic equilibrium, non-equilibrium processes may be important in quantifying the extent of mantle differentiation. Indeed, depending on the efficiency of solid-liquid phase separation, melt migration may occur on a much shorter timescale than the rate of solidification. Several mathematical models have been proposed [4–6] to evaluate the effect of non-equilibrium thermodynamics in the context of geophysical multiphase flows. Further work is required to apply these theoretical approaches to the context of a vigorously convecting magma ocean with larger initial melt fractions. However, it is important to note that non-equilibrium phase change will only increase the allowable extent of silicate-silicate fractionation that is consistent with an non-fractionated PUM; If solids do not have time to re-equilibrate, they will inevitably tend to further retain the geochemical signature of the depth where they originally form.

1.2 Trace elements partitioning

At each time step, in every grid cell, we compute the total amount of a given trace element specie i. We distribute the trace element i between the liquid and the solid phase according to its partitioning coefficient, D_i defined as:

$$D_i = \frac{x_i^l}{x_i^s}, \tag{11}$$

where x_i^l and x_i^s are the liquid and solid composition in trace element i. We use the experimentally-determined partitioning coefficient of [7] and [8]. Note that the trace elements considered here are purely passive, that is they do not affect any solid or fluid properties.

By my mass balance, we can deduce the liquid and solid composition as a function of the melt fraction:

$$x_i^s = \frac{x_0}{1 + \phi(D_i - 1)}, \tag{12}$$

where x_0 is the bulk concentration of the trace element i and ϕ is the melt fraction. Figure S3 displays the evolution of liquid and solid composition for the lower mantle and the upper mantle. Note that these plots do not involve any fluid dynamics calculation.

We distribute the trace elements according to Eq. (12) once the equilibrium melt fraction has been reached via the procedure described in Section 1.1. In Fig. S4, we compare the analytical prediction shown in Fig. S3 with compositions obtained using our fluid dynamics simulations. This allows us to benchmark the numerical implementation of trace elements partitioning. The simulations correspond to a case where phase separation is efficient ($\delta = 20 \times 10^{-3}$) such that it maximizes the effect of solid-liquid fractionation. Fig. S4 shows the frequency of occurrence of a given composition. This

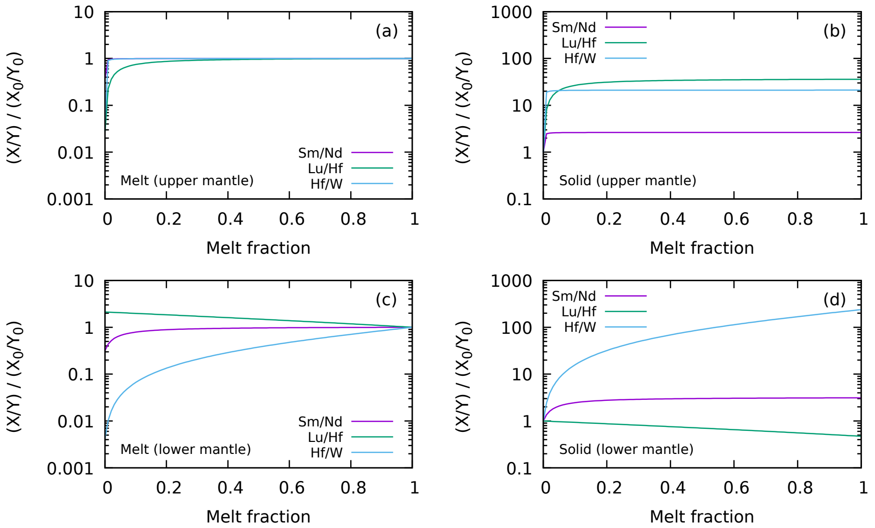

Fig. S3 Composition of the melt and solids in Sm/Nd (magenta), Lu/Hf (green) and Hf/W (cyan) as function of melt fraction. This plot does not involve any fluid dynamics calculation and severs as a consistency check for the behaviour of our numerical simulations (see Fig. S4). The trace elements ratio are normalized by their ratio in the bulk silicate Earth (BSE). (a and c) In the upper mantle, trace elements are so incompatible in the olivine that the composition departs from the BSE composition only towards the end of solidification. Solids however are very fractionated. (c and d) In the lower mantle, the trace elements considered have a better affinity for the bridgmanite. Liquids are progressively fractionated from the onset of solidification until the end of the crystallization.

snapshot in time captures the end of mantle solidification (corresponding to Figure 2 in the main text). We observed that the fluid dynamics simulations (histograms) produce compositions consistent with the partitioning model (solid and dash lines).

2 Scaling analysis and regime diagrams

2.1 Chemical differentiation near the top and bottom thermal boundary layers

Our fluid dynamics simulation revealed that chemical differentiation in magma oceans differs from what is usually thought. The canonical magma ocean model assumes that solidification and solid-liquid chemical fractionation occur at the depth where liquidus and adiabat intersect. This assumption is based on a 1D description of the temperature in a magma ocean. When considering a 2D temperature field, solids in fact precipitate at the surface of the planet in the top cold thermal boundary layer. This is because the surface temperature remains below the solidus during most of the duration of magma ocean crystallization. The crystal-rich layer is denser than the underlying mantle because both thermal and compositional density contrasts - we detailed below where

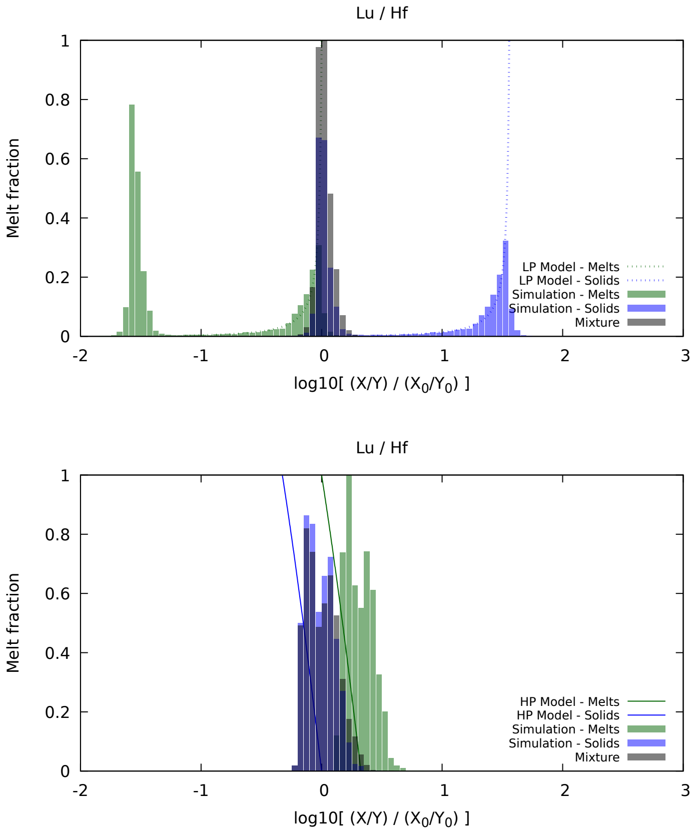

Fig. S4 Benchmark of trace elements partitioning in the fluid dynamics simulations. Analytical predictions of both melt and solid composition (green and blue lines) estimated by a simple partitioning model (see Eq.(12) and Fig. S3). (Top) Partitioning coefficients correspond to the one of olivine. (bottom) Partitioning coefficients correspond to the one of bridgmanite. Assuming a melt fraction of 10 %, we can predict the composition of the melt and solid by determining the intersection of the dash (top) or solid (bottom) lines with a horizontal line drawn at y=0.1. This is in good agreement with the frequency distribution of the simulations. However, the fluid dynamics simulations provide a more complex picture of the compositional fields, revealing a distribution of composition rather than a unique composition.

the origin of this compositional differences comes from - act in the same direction. These density contrasts drive the growth of Rayleigh-Taylor instabilities forming crystal-rich downwellings (Figure S7).

As shown in this study, crystals formed at the surface of planet play a major role in the differentiation of the mantle. Indeed, these solids experience shallow depth solid-liquid chemical fractionation governed by low-pressure mineral phases. These crystal then sink as downwellings in the deep mantle without remelting (see Section 2.3). This is the reason why the chemical evolution of

the deep mantle is expected to be significantly affected by low-pressure solid-liquid partitioning. However, to bring a fractionated composition into the deep mantle, solids and liquids must segregate from each other in the vicinity of the surface (cold) thermal boundary. Otherwise, it is the bulk composition of the liquid - that can also be fractionated at other locations - that is brought to the deep mantle.

Here, we perform a scaling analysis to compare the growth timescale of a Rayleigh-Taylor instability with that of solid-liquid segregation. First, we derive a dimensional equation to quickly estimate the timescale ratio using conservative values. Then, we reformulate it with dimensionless numbers to better relate our fluid dynamics simulation to realistic conditions.

The growth timescale for a Rayleigh-Taylor instability driven by a density anomaly of size R_0 in a domain of viscosity η scales as:

$$\tau_{RT} \sim \frac{\eta}{\Delta\rho_{RT} g R_0}. \tag{13}$$

where η is a function of viscosity, $\eta = \eta_s \exp(-30\phi)$ [9, 10]. Assuming that the compaction length is smaller that the thickness of the thermal boundary layer - we will come back the this assumption later - the timescale for solid-liquid segregation in this layer that we assume to be mostly solid, is controlled by the melt percolation velocity:

$$v_{\text{perco}} = \frac{k(\phi)}{\phi}\frac{\Delta\rho_{sl} g}{\eta_l}, \tag{14}$$

where $\Delta\rho_{sl}$ is the density difference between solid and liquid, and the function $k(\phi)$ describes the matrix permeability as function of the melt fraction. We use:

$$k(\phi) = \frac{1}{C_0} r_0^2 \phi^n, \tag{15}$$

where r_0 is the crystal size, C_0 is the tortuosity of the melt network, and n is a power exponent that captures the melt network connectivity. Using the two equations above, the timescale of solid-liquid segregation over a thickness R_0 scales as

$$\tau_{\text{seg}} \sim \frac{C_0 R_0 \eta_l}{\phi^{n-1} \Delta\rho_{sl}\ g\ r_0^2}. \tag{16}$$

Solid can segregate from the top thermal boundary layer if

$$\frac{\tau_{RT}}{\tau_{\text{seg}}} > 1. \tag{17}$$

Using Equations (13) and (16) and assuming that the density contrasts $\Delta\rho_{sl}$ and $\Delta\rho_{RT}$ are comparable yields:

$$\frac{\tau_{RT}}{\tau_{\text{seg}}} \sim \frac{\eta}{\eta_l}\frac{\phi^{n-1}}{C_0}\left(\frac{r_0}{R_0}\right)^2. \tag{18}$$

For a realistic magma ocean, we use the conservative value of $R_0 = 100$ km (today's Earth's oceanic lithosphere thickness), $r_0 = 1$ cm, $\eta_s = 10^{22}$ Pa s, $\eta_l = 10^2$ Pa s, $C_0 = 300$, $n = 3$ and $\phi = 0.15$. This yields:

$$\left(\frac{\tau_{RT}}{\tau_{\text{seg}}}\right)_{\text{Earth}} \approx 0.83. \tag{19}$$

While close to 1, this value remains a conservative estimate, because the thermal lithosphere was likely thinner during the Hadean due to significantly higher heat flux out of the Earth's interior. Additionally, Equation (18) shows that the ratio of these timescales depends on ratio of quantities that are not well-known, such as the grain size in a solidifying magma ocean, r_0, the viscosity of the melt η_l, the thickness of the upper thermal boundary layer, R_0, or the effective viscosity of the essentially solid upper thermal boundary, η.

To gain a more robust estimation of the ratio of the Rayleigh-Taylor and melt segregation timescales, we rely in the following on scaling laws to rewrite Equation (18) as a function of dimensionless numbers that describe our fluid dynamics simulations, *e.g.*, Ra and δ. Finally, this approach allows one to relate the dynamic regime observed in our fluid dynamics simulations to the one of the real Earth.

The ratio of the crystal size to thickness of the thermal boundary layer in our simulations can be estimated using the definition of the melt mobility number (see Method),

$$\left(\frac{r_0}{R_0}\right)^2 = C_0\delta^2\left(\frac{H}{R_0}\right)^2\frac{\eta_l}{\eta_s}. \tag{20}$$

Following [11] (their Equation (9.53)), the thickness of the uppermost thermal boundary layer can be related to the mantle thickness in the presence of large viscosity contrasts between the top cold layer and the hot interior:

$$R_0 \sim H Ra^{-1/3}\left(\frac{\Delta T_m}{\Delta T_R}\right)^{4/3}, \tag{21}$$

where Ra correspond to the global Rayleigh number computed with the viscosity relevant for the interior. We make a Frank-Kamenetskii approximation [e.g., 12] where ΔT_R is a temperature scale that captures the variation of viscosity in the top cold boundary layer and in the hot interior (see e.g., Equation

(9.36) in [11]),

$$\frac{\Delta T_m}{\Delta T_R} = \ln\left(\frac{\eta}{\eta_{\text{int}}}\right). \tag{22}$$

The ratio of the Rayleigh-Taylor instability growth to solid-liquid segregation timescales thus scales as:

$$\left(\frac{\tau_{RT}}{\tau_{\text{seg}}}\right) \sim \frac{\eta}{\eta_s}\phi^{n-1}\delta^2 Ra^{2/3}\left[\ln\left(\frac{\eta}{\eta_{\text{int}}}\right)\right]^{-8/3}, \tag{23}$$

where Ra corresponds to the Rayleigh number computed with the viscosity of the partially molten convective interior, η_{int}. We rewrite Ra as follows,

$$Ra = Ra_{\text{ref}}\frac{\eta_s}{\eta_{int}}, \tag{24}$$

where Ra_{ref} is a thermal Rayleigh number computed with the viscosity of the solid, η_s. This yields:

$$\left(\frac{\tau_{RT}}{\tau_{\text{seg}}}\right) \sim \frac{\eta}{\eta_s}\phi^{n-1}\delta^2 Ra_{\text{ref}}^{2/3}\left(\frac{\eta_s}{\eta_{\text{int}}}\right)^{2/3}\left[\ln\left(\frac{\eta}{\eta_{\text{int}}}\right)\right]^{-8/3}. \tag{25}$$

In our fluid dynamic simulations, $\eta \approx \eta_s$ and $\eta_{\text{int}} \approx \eta_l$ when the melt fraction is far from the rheological critical melt fraction (see MT Equation (9)). We use $\delta = 1.12 \times 10^{-2}$ for our reference case (described in the main text), $Ra_{\text{ref}} = 10^6$, $\frac{\eta_s}{\eta_l} = 10^3$, $\phi = 0.15$ (melt fraction in the vicinity of the boundary layer), $n = 2$ (as in our fluid dynamic model, see [13]), and find for our reference case:

$$\left(\frac{\tau_{RT}}{\tau_{\text{seg}}}\right)_{\text{simulations}} \approx 0.11, \tag{26}$$

indicating that solid-liquid differentiation in the thermal boundary top thermal boundary layer in our reference simulation is expected to occur, but may not be significant. We performed other simulations with $\delta = 3 \times 10^{-2}$, $Ra = 10^8$, $\frac{\eta_s}{\eta_l} = 10^2$, yielding to,

$$\left(\frac{\tau_{RT}}{\tau_{\text{seg}}}\right)_{\text{simulations}} \approx 0.88, \tag{27}$$

which is closer to unity. In our simulations, the maximum viscosity ratio between solids and liquid is 10^4. However, we achieve a realistic ratio for these two timescales (and thus similar dynamic behavior) by artificially increasing the size of the crystals (see Figure S5a). By doing so, we compensate for the smaller viscosity contrast, ensuring that the dimensionless melt mobility

number is closer to Earth's conditions. As a result, phase segregation velocities become comparable to convective velocities driven by Stokes flow (see Section 2.2 and [13]).

For Earth-like conditions, we have $\eta = \eta_s \exp(-30\phi)$ [9, 10], but the ratio of the solid viscosity to the one of the interior - that controls the thickness of the upper thermal boundary layer - as well as the melt mobility are subject to large uncertainties. To anticipate the expected range of uncertainties, we plot in Figure S5b, the ratio of the Rayleigh-Taylor timescale and solid-liquid segregation as a function of the melt mobility number, and the viscosity contrast between the cold the surface and the interior. For completeness, we assume that the mushy interior is 10^5 to 10^{15} times less viscous than the essentially solid upper thermal boundary layer. Assuming crystal sizes, r_0 between 1 mm and 10 cm, and a solid / liquid viscosity contrast between between 10^{14} and 10^{20}, we expect the mobility number for a real Earth to range between 10^{-5} and 10^2 (see Figure S5a). For these values, there is a large parameter space where the ratio of the Rayleigh-Taylor timescale is several orders of magnitude larger than the timescale for solid-liquid segregation. This is the reason why we anticipate solid-liquid segregation within the top thermal boundary layer in our simulations. This process is expected to be more significant for the Earth - for sufficiently large melt mobility numbers and viscosity contrasts between the upper thermal boundary layer and the mushy interior - as indicated by this scaling analysis.

Finally, we tested our working hypothesis, that consists in neglecting compaction in the thermal boundary layer, by comparing the thickness of the upper thermal boundary layer (Equation (21)) with the compaction length (Equation (3.3) [14])—which is not directly equivalent to our melt mobility number:

$$L_{\text{comp}} = r_0 \sqrt{\frac{\phi^n}{C_0} \frac{\eta_s}{\eta_l}}. \tag{28}$$

Figure S6 shows R_0/L_{comp} as a function of the viscosity ratios. Values larger than one indicate that the thermal boundary layer is thicker than the compaction length, implying that compaction can be neglected. For plausible solid/liquid viscosity ratios, this analysis shows that the viscosity contrast between the thermal boundary layer and the mushy interior cannot be too large (*i.e.*, $< 10^{14}$) for compaction to be negligible in the upper thermal boundary layer.

As depicted in Figure S7, the velocity field for the liquid phase (shown by red arrows) and for the solid phase (shown by blue arrows) near the top boundary layer in our reference simulation ($\delta = 1.12 \times 10^{-2}$, $Ra = 10^9$, $\frac{\eta_s}{\eta_l} = 10^3$). The figure illustrates that the motion of the two phases is primarily driven by phase averaged thermo-chemical density contrast without phase separation. However, we observe that in some regions the liquid velocity is deflected upwards relative to the solid velocity, which is attributed to the upward flux of melt driven by the density contrast between the solid and liquid at shallow depths. This is consistent with our scaling analysis that indicates a ratio of

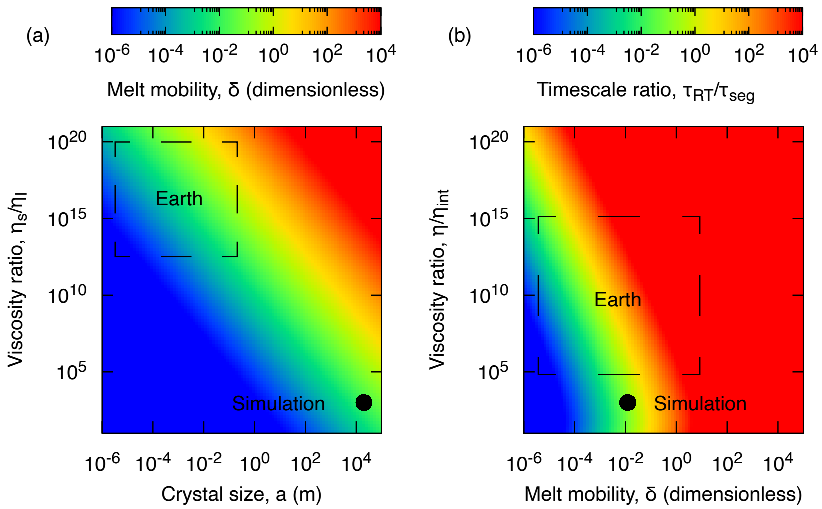

Fig. S5 (a) Melt mobility number as a function of crystal size and viscosity ratio between the liquid and solid phase. (b) Ratio of the Rayleigh-Taylor to the solid-liquid segregation timescales as a function of melt mobility number and the viscosity ratio between the upper thermal boundary layer and the interior.

the Rayleigh-Taylor growth timescale and solid-liquid segregation smaller but close to 1.

Importantly, the solid-liquid segregation mechanism near the top boundary layer leads to solids in downwelling currents that are enriched in FeO, as shown in Figure S7. This is at odds with what one would expect from the values of partitioning coefficients. These solids should be consistently depleted in FeO compared to liquids. However, any crystal forming near the cold thermal boundary tends to settle, propelling FeO-rich melt upwards by mass conservation. This FeO-rich melt approaches the cold thermal boundary, cools down by diffusion, and eventually solidifies. As a result, the cold downwellings are enriched in FeO, making them thermally and chemically negatively buoyant, as seen in Figure S9.

The symmetric version of the processes described above also occurs in the deep mantle during the formation of upwelling currents at the (hot) bottom thermal boundary (Figure S10). The main difference is that in the deep mantle, melts segregate downwards due to a density crossover between the melt and solid at approximately mid-mantle depth. Near and within the hot thermal boundary layer, solid materials heat up by diffusion and may remelt. Any remaining crystal tend to float, driving FeO-rich melt downwards. Consequently, this denser FeO-rich melt accumulates at the core-mantle boundary (CMB), forming the basal magma ocean (Figure S10).

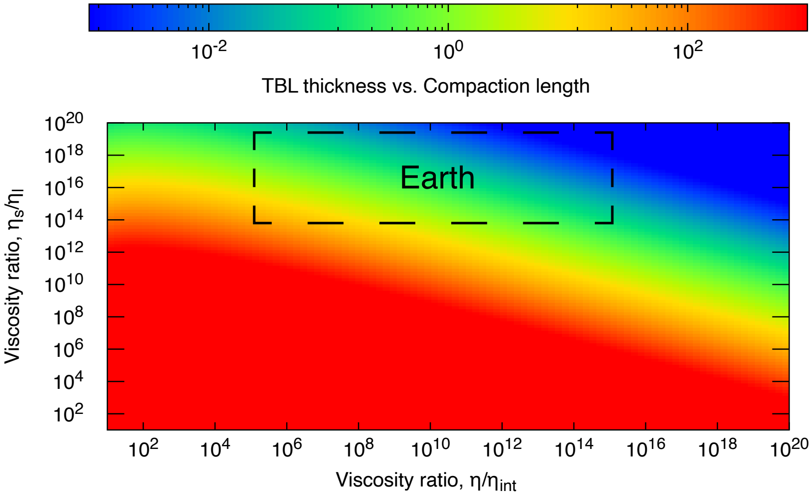

Fig. S6 Ratio of the upper thermal boundary layer (TBL) thickness and the compaction length. Ratios larger than 1 indicate that compaction can be neglected.

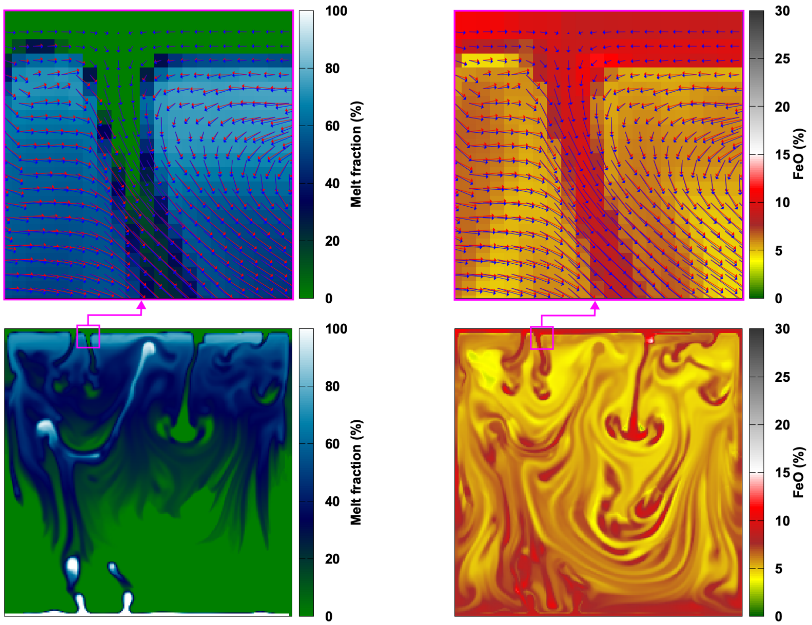

Fig. S7 Melt (red) and solid (blue) velocity fields in the vicinity of the top thermal boundary region. In some regions the liquid velocity is deflected upwards relative to the solid velocity, which is attributed to the upward flux of melt driven by the density contrast between the solid and liquid at shallow depths. The top shows closed up views the of the fluid parcels highlighted in magenta in the bottom row.

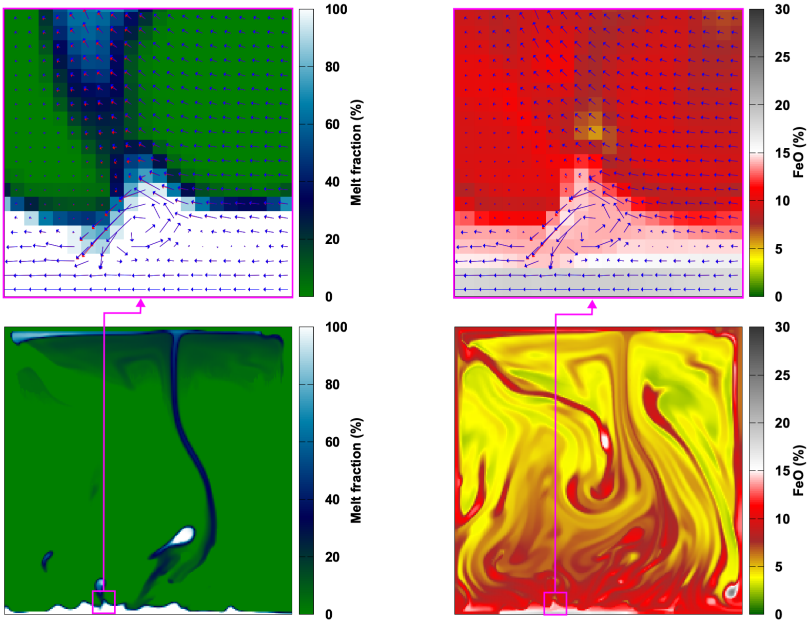

Fig. S8 Same as Figure (S7) but for the lower mantle in the vicinity of the basal magma ocean (BMO). In several regions the liquid velocity (red arrows) is deflected downwards relative to the solid velocity (blue arrows), which is attributed to the downwards flux of melt driven by the density contrast between the solid and liquid in the lower mantle. The top panels show closed up views the of the fluid parcels highlighted in magenta in the bottom row.

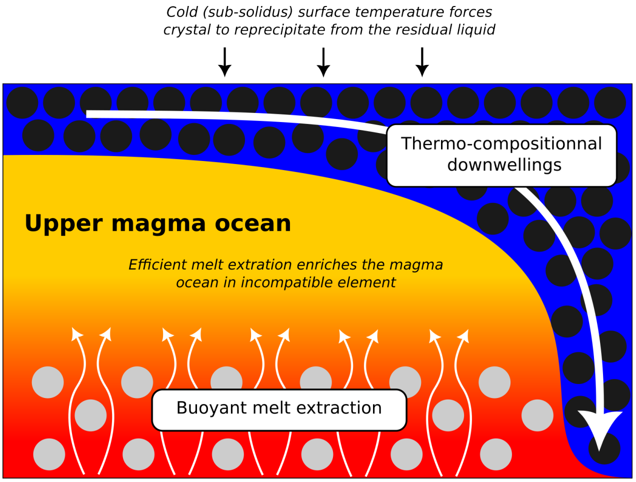

Fig. S9 Schematic of fluid dynamics processes involved in the chemical differentiation near the top thermal boundary layer. This is the interpretation of the velocity field of the fluid dynamics simulations shown in Figure S7.

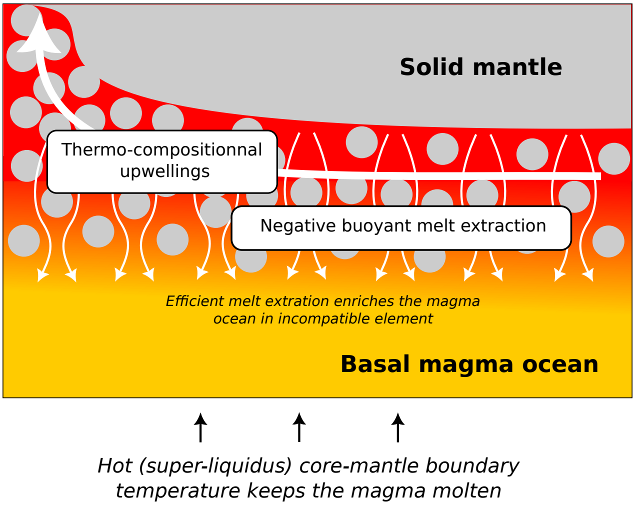

Fig. S10 Schematic of fluid dynamics processes involved in the chemical differentiation near the bottom thermal boundary layer.This is the interpretation of the velocity field of the fluid dynamics simulations shown in Figure S8.

2.2 Solid-liquid phase separation *vs.* entrainment by thermal convection

In the literature, MO thermal evolution models, that track the evolution of the MO melt fraction with time, are generally decoupled from mechanical models of solid-liquid segregation, that track where crystals accumulate. Historically, this choice was motivated by the fact that the first MO thermal models were mostly designed to investigate primordial atmosphere degassing, not mantle differentiation. In these work, the actual structure of the planet interior, i.e., the spatial distribution of crystals, was of secondary importance [15–18]. In fact, if the adiabat is steeper that the liquidus, and crystals remain denser than the melt at all depth, we should always expect an upper magma ocean on the top of solid cumulates. In that case, the location of crystals, e.g., averaged crystal fraction in the magma ocean and the amount of residual liquid in the cumulates, play a relatively minor role on the MO thermal evolution. The basal magma ocean hypothesis [19] changed that paradigm as the presence of a solid shell at mid-mantle depth may thermally decouple the upper magma ocean from the lower magma ocean. In that case, the location where crystals accumulate becomes crucial for the thermal evolution of the magma ocean. [20] proposed the first 1D thermal evolution model capable of accounting for a solid layer at mid-mantle depth. As no crystal spatial distribution is prescribed a priori, substantial numerical challenges arises from the time-integration of the energy equation with a large dynamic range of energy fluxes (see also [21]). However, in this kind of modelling, the temperature (or entropy) is not advected independently from the crystal fraction (but it may be done in theory). Assuming thermodynamic equilibrium, this implies that the spatial

distribution of crystals mimics exactly the relation between temperature and melting curves. In this model, the formation of a basal magma ocean is thus only dictated by temperature, not where crystals go. Here, we aim to propose an alternative scenario where the formation of the basal magma ocean is not dictated by the temperature profile, but by where crystals go. To do that, we must advect the crystal fraction independently from the temperature.

Independently from magma ocean thermal evolution models, the physics solid-liquid phase separation in magma oceans has received considerable attention since the development of the lunar magma ocean hypothesis [22, e.g.,]. Indeed, the physics of solid-liquid phase separation is crucial for the issue of mantle differentiation. Because large melt fractions are required for solid feldspar to segregate from the residual magma to form the primary lunar crust [23], models of magma ocean differentiation mostly focused on the physics of solid-liquid segregation at high melt fraction, *i.e.*, crystal settling and suspension [20, 23–32].

Here we focus on magma ocean differentiation beyond the rheological transition, *i.e.*, once the mantle reaches a solid-state like dynamics (with crystal fractions of about 50 %). Major differences are expected between magma ocean differentiation at low crystal fraction and high crystal fraction. First, the effective thermal Rayleigh number in a mushy magma ocean ($< 10^{15}$) is expected to be substantially lower than that of a fully molten magma ocean ($> 10^{27}$), making the dynamics of mushy magma oceans more closely accessible with current computational capabilities. In this case, solid-liquid segregation is not governed by crystal settling and re-entrainment but by melt percolation, matrix compaction, and shear deformation. Second, during the mushy magma ocean stage, strong feedbacks between the mechanics of solid-liquid segregation and phase change may occur because phase change produces compositional differences that affect solid-liquid density contrast in turn affecting local composition and thermodynamic equilibrium.

To cross-check the relevance of our numerical model that accounts for phase change, chemical fractionation, and solid-liquid viscosity contrast, we analytically predicted the transition between fractional and batch solidification using a simple approach. To do so, we followed previous studies that estimated the transition between the two magma ocean stages regimes mentioned above by comparing the magnitude of velocities associated with thermal convection and phase separation [13, 33]. This simple approach provides an upper bound for the transition between batch and fractional crystallization (see below). Crystals settling at the bottom of the magma ocean (where the vertical component of the convective velocities decreases) can still occur even if crystal velocities are substantially smaller than the magnitude of convective velocities [24, 27, 34]. Therefore, despite its simplicity, comparing the average convective velocity and phase separation appears relevant for validating our numerical approach. We describe below how these two velocities can be extracted from our mathematical description of the magma ocean multi-phase physics.

Mechanical differentiation is governed by competition between solid-liquid phase separation and thermal convection as we consider that the timescale of solidification is not a limiting factor. Indeed, in a sluggish state, the cooling timescale of the magma ocean is larger than the timescale of melt migration. We thus consider two end-member cases:

- **Fractional solidification:** Solid-liquid phase separation generates a compositional layering much faster than thermal convection can stir this layering. This regime promotes fractional crystallization where melt of distinct composition is perfectly extracted from the residual matrix before any large-scale convective motion can occur.
- **Batch solidification:** Thermal convection mechanically stirs any new formed layering faster than phase separation generates this compositional layering. This regime promotes batch crystallization where the mantle is constantly homogenized during solidification.

To estimate the mean velocity of convective motions driven by thermal density contrast, $v_{\text{mean}}^{\text{conv}}$, we use a scaling law of the form [35].

$$v_{\text{mean}}^{\text{conv}} = 0.16 Ra^{\frac{3}{5}}. \tag{29}$$

To estimate the average phase separation velocity, $v_{\text{mean}}^{\text{sep}}$, we use MT Equation (10) assuming that melt extraction is solely driven by density contrasts between melt and solid, and we consider a melt fraction of 50%. We obtain:

$$v_{\text{mean}}^{\text{sep}} = \frac{1}{4} Ra \delta^2. \tag{30}$$

Importantly, the phase separation velocity is a function of the melt mobility number that accounts for both the melt viscosity and grain size. Fig. S11 maps regions in the (Ra, δ) space, where batch crystallization or fractional crystallization are expected to occur. The transition between the two regimes corresponds to $v_{\text{mean}}^{\text{conv}} = v_{\text{mean}}^{\text{sep}}$. Despite its simplicity, this prediction is in good agreement with the results from our numerical simulations (see Fig. S11). In our study, we conducted simulations that are in the vicinity of the boundary between the two regimes (Fig. S11). For a maximum thermal Rayleigh number of 10^9, we observe inefficient differentiation for $\delta = 5 \times 10^{-3}$ (Fig. S12) and substantial differentiation for $\delta = 20 \times 10^{-3}$ (Fig. S13). As expected, we do still observe differentiation when $v_{\text{mean}}^{\text{conv}} > v_{\text{mean}}^{\text{sep}}$, but we do not observe differentiation when $v_{\text{mean}}^{\text{conv}} < v_{\text{mean}}^{\text{sep}}$. This is consistent with our prediction that provides an upper bound for the transition between batch and fractional crystallization as discussed above. This diagram allows to explore magma ocean dynamics in regimes that are accessible with current numerical limitations, but where the critical issue of batch *vs.* fractional crystallization can still be investigated in a quantitative manner (Fig. S11 and S16).

The regime diagram can also be used to predict the crystal sizes in magma ocean (Fig. S16). If we first consider that melt viscosity is about 10^4 Pa s, the expected maximum thermal Rayleigh number is 10^{24}. Assuming that the

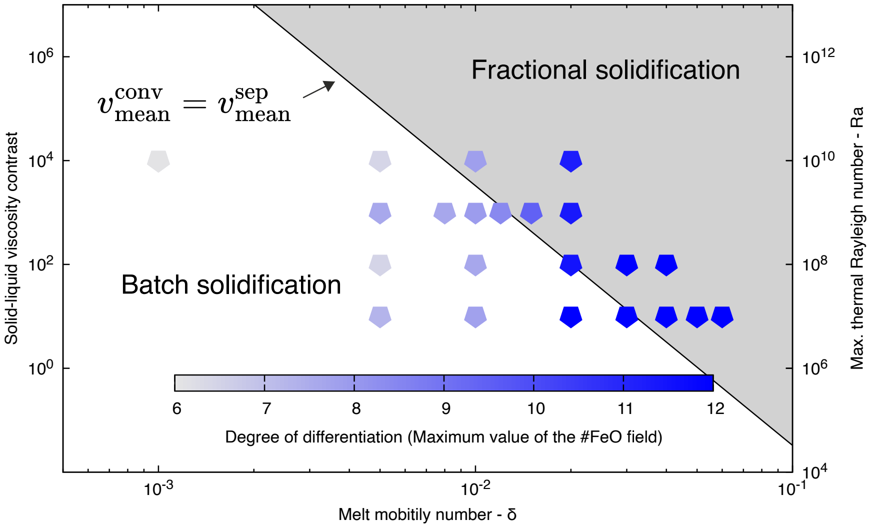

Fig. S11 Regime diagram in a *Ra* (thermal Rayleigh number) - δ (melt mobility) dimensionless space. 22 fluid dynamics simulations (colored diamonds) are used to quantify the amount of chemical differentiation in magma ocean. The maximum concentration of FeO serves as a proxy to measure the degree of chemical differentiation. These numerical simulations that capture several aspects of the magma ocean solidification, are in good agreement with our analytical predictions based purely on mechanical consideration (see text).

Earth's magma ocean solidifies in the intermediate regime, δ must be around 10^{-5}, this sets a maximum crystal size to 1 μm. If we consider a much lower melt viscosity value of about 1 Pa s [36], this further decreases the maximum crystal size to 0.01 μm. These values are consistent with previous estimates [27, 28, 37, 38].

Finally, it is important to identify the driving forces for solid-liquid segregation in our simulations. Although body forces, specifically the density contrast between solid and liquid, are generally the main drivers of solid-liquid segregation in the mushy mantle, this is not universally the case across the convective domain (see Figure S14). We observe that deviatoric stresses (the last term on the right side of MT Equation (10)) drive strongly phase separation at the crystallization front, where shear deformation and a large viscosity contrast occur within relatively small regions. Additionally, we note that compaction plays a non-negligible role in regions where there the melt fraction gradient is high. However, this effect is confined to very small regions, consistent with the small values of compaction length used in our simulations.

2.3 Crystal remelting during their descent

Solid silicates that form at a planet's surface retain signatures of low-pressure mineral-melt partitioning. In this context, we estimate the extent of remelting expected in a crystal-rich parcel of fluid as it descends into the warmer mantle. These crystal-rich blobs originate as Rayleigh-Taylor instabilities within

Parameters	Symbol	Value	Unit
Mean density	ρ_0	4500	kg m^{-3}
Thermal expansivity	α	10^{-5}	K^{-1}
Gravity acceleration	g	9.81	ms^{-2}
Magma ocean thickness	H	3000	km
Thermal diffusivity	κ	10^{-6}	m^2 s^{-1}
Solid-liquid isochemical density contrast at the surface	-	1400	kg m^{-3}
Solid-liquid isochemical density contrast at the CMB	-	100	kg m^{-3}
Maximum chemical density contrast at the surface	-	500	kg m^{-3}
Maximum chemical density contrast at the CMB	-	1500	kg m^{-3}
Thermal capacity	C_p	10^3	J K^{-1} kg^{-1}
Latent heat of solidification	L	1000	kJ kg^{-1}
Solid viscosity	η_s	10^{22}	Pa s
Liquid viscosity	η_l	10^0	Pa s
Compaction viscosity	η_c	10^{22}	Pa s
Crystal size	r_0	10^{-6}- 10^{-2}	m
Surface temperature	-	500	K
Core temperature	-	5000	K
Adiabatic temperature increase over the MO thickness	-	1800	K
Super-adiabatic temperature jump over the MO thickness	ΔT_m	2700	K
Mg-rich end-member melting temperature at the CMB	T^c_{MgO}	4700	K
Fe-rich end-member melting temperature at the CMB	T^c_{FeO}	3500	K
Mg-rich end-member melting temperature at the surface	T^s_{MgO}	2054	K
Fe-rich end-member melting temperature at the surface	T^s_{FeO}	1359	K

Table S1 Values of the physical parameters used in this study.

Parameters	Symbol	Earth's MO [1]	This Study [2]
Solid-state thermal Rayleigh number	Ra	3.22×10^6	10^6
Solid-liquid viscosity contrast	-	10^{22}	10^3
Surface phase Rayleigh number	Rp^{surf} / Ra	11.523	11.523
CMB phase Rayleigh number	Rp^{cmb} / Ra	0.823	0.823
Exponent[3] in $R_p(z)$	γ	-	6
Surface comp. Rayleigh number	Rc^{surf} / Ra	4.115	4.115
CMB comp. Rayleigh number	Rc^{cmb} / Ra	12.346	12.346
Stefan number	S_t	0.37	0.37
Melt mobility number[2]	δ	10^{-3}-10^1	5×10^{-3} - 20×10^{-3}
Compaction viscosity	ζ	1	1
CMB temperature	-	1.0	1.0
Liquidus at the CMB	-	0.88	0.88
Solidus at the CMB	-	0.44	0.44
Liquidus at the surface	-	0.57	0.57
Solidus at the surface	-	0.31	0.31
Liquidus binary loop exponent	m	-	1.7
Solidus binary loop exponent	n	-	1.1

Table S2 Dimensionless quantities used in this study. [1] These values are computed using the parameters presented in Table 2.2. [2] Those values are constrained by current computational limitations. The appropriate solid-liquid viscosity contrast and the melt mobility number cannot be reproduced in our model. A reasonable solid-liquid viscosity contrast is currently out of reach in this kind of fluid dynamics simulations. Even though a realistic melt mobility number cannot be used in our simulations, it is re-scaled in relation to the thermal Rayleigh number so that the competition between entrainment by thermal convection and solid-liquid phase separation remains relevant in our simulations [13]. [3] The depth-dependent iso-chemical Rayleigh number is parameterized as $R_p(z) = \frac{R_p^s - R_p^{\mathrm{cmb}}}{1-e^{-\gamma}}\left(e^{-\gamma z} - 1\right) + R_p^{\mathrm{surf}}$, where z is the dimensionless depth.

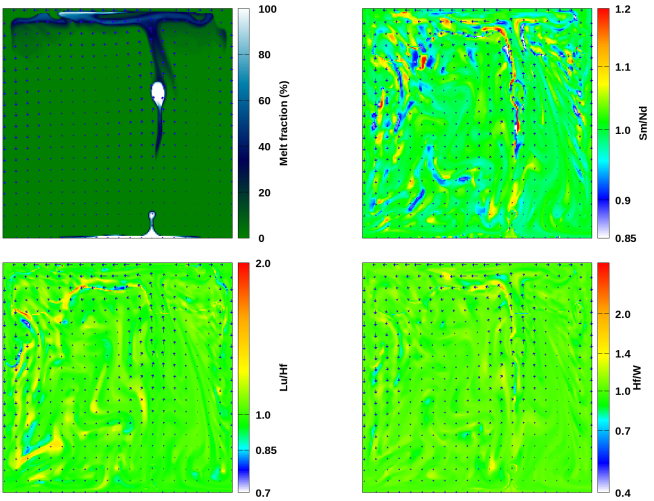

Fig. S12 Compositional fields near the end of MO solidification in the case where phase separation is inefficient ($\delta = 5 \times 10^{-3}$) at $Ra_{\max} = 10^9$), see Fig. S11

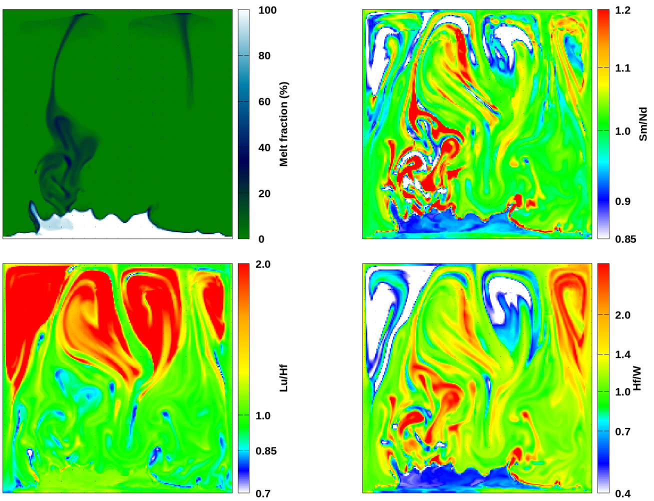

Fig. S13 Compositional fields near the end of MO solidification in the case where phase separation is efficient ($\delta = 20 \times 10^{-3}$) at $Ra_{\max} = 10^9$, see Fig. S11).

Element	D_i - Lower mantle [a]	D_i - Upper mantle [b]	Abundances in BSE (in ppb) [c]
Sm	0.05	1.1×10^{-2}	406
Nd	0.016	4.2×10^{-4}	1250
Lu	0.79	3.9×10^{-2}	67.5
Hf	1.6	1.1×10^{-3}	283
W[d]	0.007	5.2×10^{-5}	29

Table S3 Trace elements partitioning coefficients. [a] Lower mantle values [7]. [b] Upper mantle values [8]. [c] We use the model of [39]. [d] To our knowledge, solid-liquid partitioning of W in silicates has not been measured experimentally. Therefore, we use the partitioning coefficients of Th that has potentially a similar behavior [40].

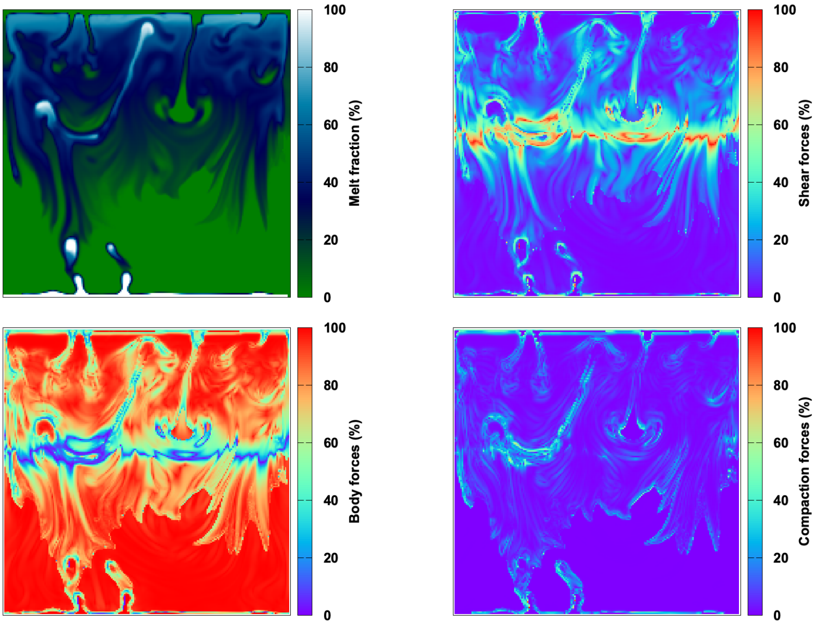

Fig. S14 Snapshot of driving forces for solid-liquid phase separation for the simulations shown in Figure S7 and S9.

the top thermal boundary layer of the mantle. Following the mathematical model proposed by [41], we describe melt production in an descending spherical blob, assuming that the blob's temperature remains between the solidus and liquidus. The conservation of energy within the blob is expressed as (see Equation (5) in [41]):

$$\rho C_p \frac{\partial T}{\partial t} + \rho L \frac{\partial \phi}{\partial t} - \alpha T \frac{\partial p}{\partial t} = k \boldsymbol{\nabla}^2 T, \tag{31}$$

Here, the first term on the left side accounts for the internal heat change in the blob, the second term represents the energy associated with phase change, and the third term reflects the energy variations due to adiabatic compression (p represents the lithostatic pressure experienced by the blob). These energy sources are balanced only by thermal diffusion on the right side of the equation, where $k = \kappa \rho C_p$ denotes the thermal conductivity.

With the temperature held between the liquidus and solidus, temperature and melt fraction are inherently linked by the phase diagram under the assumption of thermodynamic equilibrium:

$$\phi = f(T) \text{ for } T_s < T < T_l. \tag{32}$$

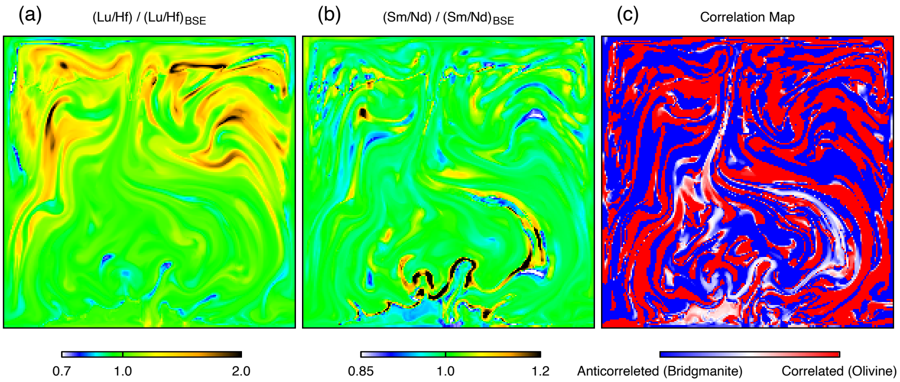

Fig. S15 Sm/Nd (a) and Lu/Hf (b) compositional fields near the end of MO solidification shown in Fig. 2 of the main text. We plot in (c) a correlation map of the Sm/Nd and Lu/Hf fields that allows to quickly visualize the origin (*e.g.*, depth of formation) of these compositional anomalies. Correlation fractionnation of Sm/Nd and Lu/Hf ratios (red) is a signature of olivine fractionation, while anticorrelation (blue) is a signature of bridgmanite fractionation.

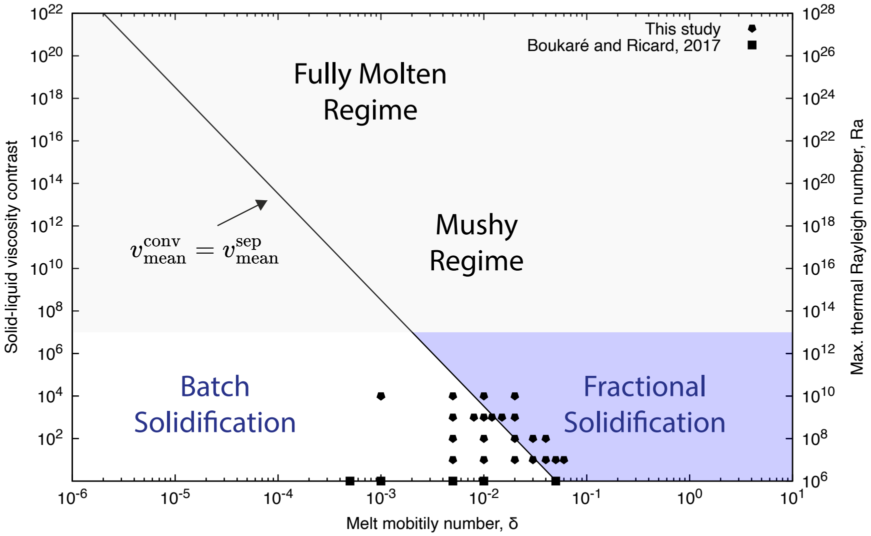

Fig. S16 Regime diagram of magma ocean dynamics. Large viscosity contrasts between solid (10^{22} Pa s) and liquid (1 Pa s) challenges our understanding of magma ocean evolution. During solidification magma ocean covers a broad range of fluid dynamic regime, ranging from fully molten turbulent convection to solid-state convection. Convective vigor with a thermal Rayleigh number larger than 10^{13} is currently not accessible via numerical simulations. However, the question whether magma ocean solidifies in a batch or fractional scenario can still be addressed in convective regimes accessible with current numerical resources by adjusting the melt mobility number.

For simplicity, we assume a linear relationship between melt fraction and temperature (which is a plausible working hypothesis):

$$\frac{\partial \phi}{\partial t} = \frac{\partial \phi}{\partial T}\frac{\partial T}{\partial t} = a\frac{\partial T}{\partial t}, \tag{33}$$

where a is a constant. Assuming the spherical blob descends at a constant velocity W, the lithostatic pressure experienced by the blob during its descent, $p(t)$, is given by:

$$p = \rho g W t \tag{34}$$

We can now rewrite Equation (31) as:

$$\rho(Cp + L\ a)\frac{\partial T}{\partial t} = k\boldsymbol{\nabla}^2 T + \alpha T \rho g W, \tag{35}$$

We non-dimensionalize Equation (35) using the timescale H/W and R_0, the radius of the spherical blob, as the length scale, yielding:

$$\frac{\partial \theta}{\partial \tau} = \frac{1}{Pe}\boldsymbol{\nabla}^2\theta + A, \tag{36}$$

where θ is the dimensionless temperature, τ is the dimensionless time, and the Péclet number, Pe, is the ratio of diffusion to advection timescales:

$$Pe = \frac{W R_0^2}{\kappa_e H}, \tag{37}$$

where $\kappa_e = k/[\rho(Cp + L\ a)]$ is an effective diffusivity that accounts for latent heat release. The dimensionless number A corresponds to a dimensionless adiabatic gradient:

$$A = \frac{\alpha T_0 g W}{Cp + L\ a}. \tag{38}$$

Equation (36) shows that temperature (and thus melt fraction) variation is predominantly controlled by the adiabatic gradient when the Péclet number is large (indicative of rapid descent without significant heat exchange with the surrounding mantle). This adiabatic compression is unfavourable to remelting as pressure increases (due to the slopes of liquidus and adiabat) and preserves solids formed at shallow depths in solid form. This process can be seen as the opposite version of decompression melting.

Next, we evaluate the Péclet number for a mushy mantle. We start by assuming that the blob descends at Stokes velocity, primarily driven by thermal density variations (note that in our simulations, compositional factors actually increase the blob's density, therefore this is a conservative estimate):

$$W = \frac{\rho_0 \alpha \Delta T_m g R_0^2}{2\eta}, \tag{39}$$

Springer Nature 2021 LaTeX template

where η corresponds to the average fluid viscosity within the mushy mantle, and the temperature difference between the upper thermal boundary layer and the interior is $\Delta T_m/2$. Upon substitution of Equation (39) into Equation (37), we obtain:

$$Pe = \frac{\rho_0 \alpha g \Delta T_m R_0^4}{2\eta \kappa_e H}. \tag{40}$$

As seen in the equation above, estimating Pe requires the knowledge of the blob size, R_0. Assuming the blob matches the size of the upper cold thermal boundary layer, we apply Rayleigh–Nusselt scaling laws (Equation (21)) to link R_0 to the mantle thickness, H. We assume that the melt fraction varies from 0 to 100 % over a temperature range of 300 K [1, 2], therefore we define $a = \frac{1}{300}\ K^{-1}$, implying that $\frac{\kappa}{\kappa_e} \sim 1$. Combining Equations (40), and (21) leads to:

$$Pe \cong \frac{1}{2} Ra^{-1/3} \left[\ln \left(\frac{\eta_s}{\eta} \right) \right]^{16/3}. \tag{41}$$

where Ra is the global thermal Rayleigh number of the partially molten interior. If this requirement is met ($i.e$, $Pe \gg 1$), solids forming in the top boundary layer cannot remelt via heat diffusion during their descent (though they may remelt at the core-mantle boundary when the vertical advective velocity tapers). These solids will carry low-pressure chemical fractionation signatures into the deep mantle.

Since the thermal Rayleigh number also depends on the effective viscosity of the partially molten interior, we plot in Figure S17 the Péclet number as a function of the viscosity contrast between the surface and the partially molten interior, assuming a reference thermal Rayleigh number of $Ra_{\mathrm{ref}} = 10^6$ computed with the viscosity $\eta_s = 10^{22}$ Pa s,

$$Ra = Ra_{\mathrm{ref}} \frac{\eta_s}{\eta}. \tag{42}$$

Figure S17 shows that crystal remelting is not expected in a mushy magma magma ocean when the viscosity contrast between the solid surface and the mush is lower than approximately 10^{21}. This criterion is also satisfied in our fluid dynamics simulation, where the maximum viscosity contrast between solid and liquid is 10^4.

It is important to note that this scaling analysis is primarily based on a scaling law originally developed for mantle convection with strong viscosity contrasts driven by thermal variations. In the future, it would be interesting to test the validity of this scaling law for multi-phase systems, which also exhibit a wide range of viscosities due to changes in melt fraction.

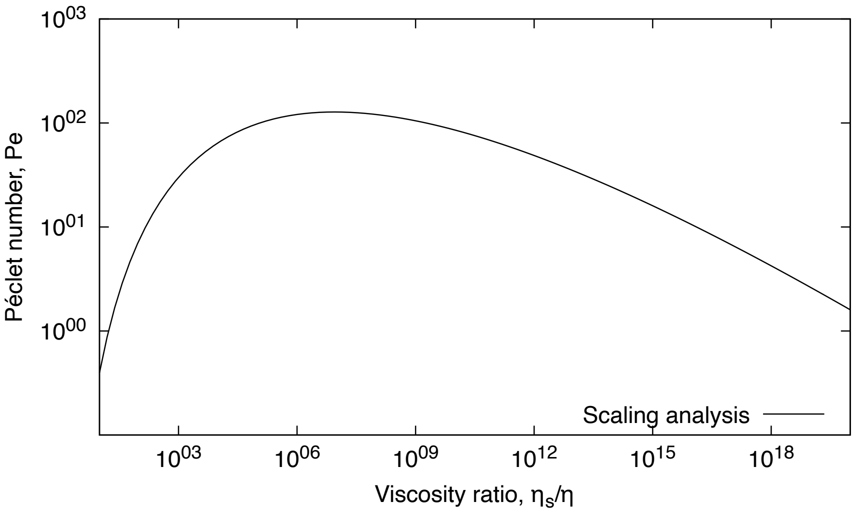

Fig. S17 Regime diagram for crystal remelting during their descent based on available scaling laws. A Péclet number value larger than one implies no solids remelting during their descent.

3 Additional simulations and videos

3.1 Solidification with a super-isentropic melting curve in the deep mantle

We conducted a simulation where the melting curves first intersect the adiabat at mid- mantle depth [42]. The dynamics is very similar to the case where the liquidus first intersects the adiabat at the CMB. It must be noted that crystal-rich downwellings that form at the surface do not accumulate at mid-mantle depth as they remain thermally negatively buoyant. They pursue their descent and remelt in the deep mantle. This further underlines that the crossing of the liquidus and adiabat does not play a major role in the style of solidification of the magma ocean (Fig. S18).

3.2 Preservation of a primordial undegassed reservoir

To quantify the amount of degassing in our simulations, we follow the approach used in [43] where we relied on Lagrangian particles to track the minimum depth reached by the magma ocean for the entire duration of our simulation and compare them with exsolution depths for relevant volatiles. Each tracer therefore represents a magma ocean parcel. If a given tracer does not reach the exsolution depth, it remains undegassed, *i.e.*, it retains it primordial noble gas signature and volatile content. In contrast, as soon as a given tracer reaches the exsolution depth it will exsolve all its super saturated volatiles and loose them to the atmosphere. For simplicity, we assume that the magma ocean is undegassed at the beginning of our simulations. We use Ra=10^6, a solid-liquid

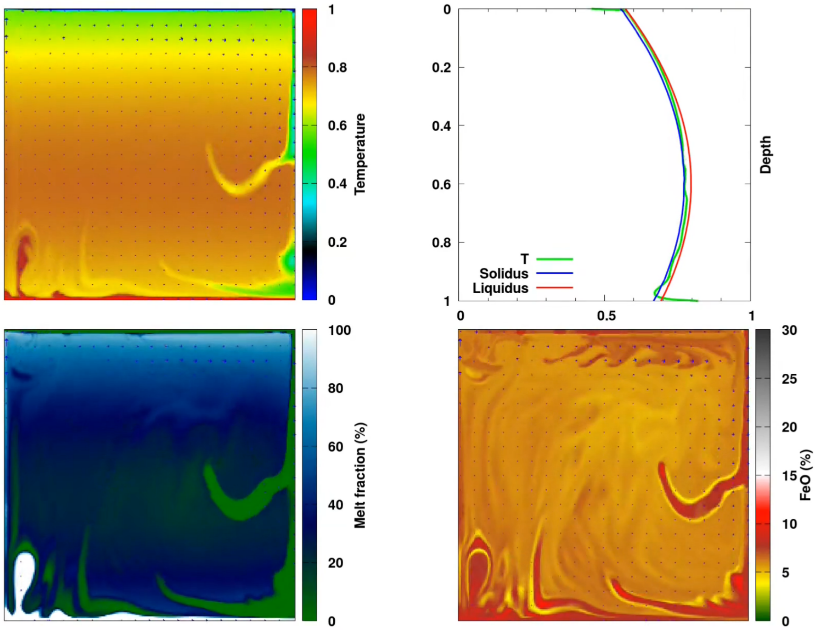

Fig. S18 Case where the melting curves first intersect the adiabat at mid-mantle depth [42].

viscosity contrast of 10^3 and $\delta = 15 \times 10^{-3}$. Over the duration of the numerical experiments, the tracers travel an average vertical distance of 15 times mantle thickness. The results are show in Fig. S19 which displays the amount of volatiles that remains in the magma ocean at the end of our simulation for four different exsolution depths. Our results are consistent with those of [43] (performed in the case of a fully molten magma ocean) that show that the exsolution depth must be significantly large to degas the entire mantle on short time interval. For an exsolution depth of 3 km, which is already is an extreme upper limit for Earth conditions [43], we observe only a negligible amount of degassing in our simulations (see Fig. S19). These results are expected to depend on the transit times performed by the magma ocean (*i.e.*, the number of times a magma ocean "travels" from one horizontal boundary to the other) [43]. In the present simulations, the number of transit is 15.

Note that in the case considered here the coexistence of the liquid and solid phases along with the possibility of multiple melting and solidification processes limits the applicability of the tracer approach proposed in Samuel and Salvador (2023). For this reason, our results are only a simplification of the reality that reflects the weak ability of mantle convective dynamics to exsolve its volatiles species. A more thorough investigation is therefore required to more finely assess the outgassing capability of mushy magma oceans to exsolve their volatiles but this could require a complete re-implementation of the numerical model to properly track dissolved volatiles in space and time

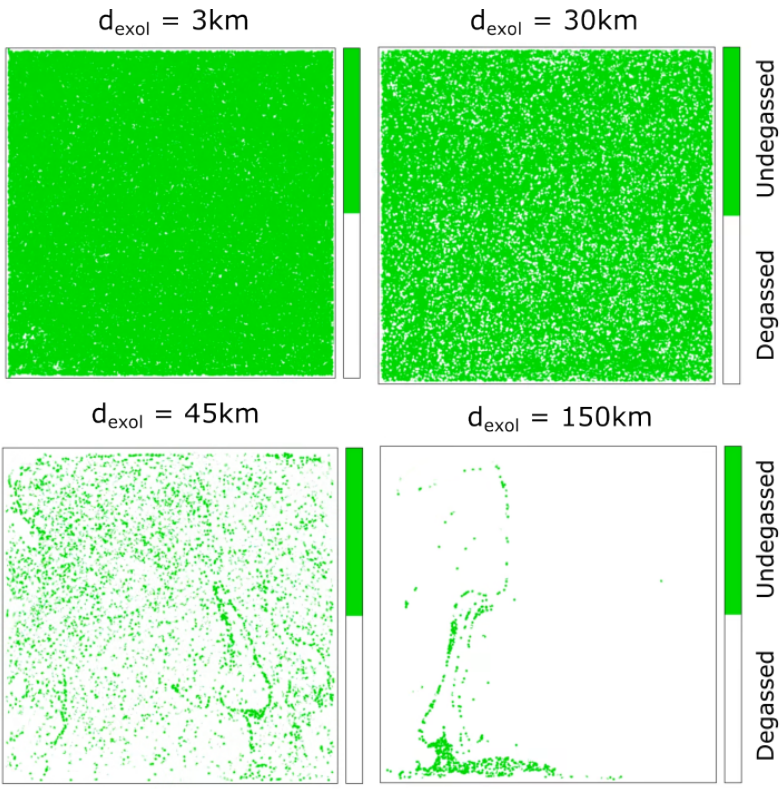

Fig. S19 Amount of material exsolved after 15 transit times for four different exsolution depths for the case displayed in Fig. 1. The exsolution depth must be significantly large (far beyond plausible values) to allow complete degassing of the Earth's mantle during magma ocean solidification. For an exsolution depth of 3 km, which is already an extreme upper limit [43], we observe a negligible amount of degassing in our simulations.

and across melting and solidification processes, which goes beyond the main purpose of our current study.

Regardless, the preservation of a primordial and undegassed mantle reservoir has recently been questioned [44], which concluded that while the rare gazes isotopic signals collected at hotspot locations may originate from separate reservoirs, these sources may be associated with low concentrations in volatiles.

Overall, these considerations suggest that our results are not incompatible with constraints arising from geochemical analyses of lava samples.

3.3 Videos

The videos of numerical simulations described in the main manuscript can be downloaded by using the following links.

Springer Nature 2021 LaTeX template

We provide three files that correspond to the cases displayed in the Figures 1 (fig1_video.mp4) and 2 (fig2_video.mp4) of the main text, and the additional case that explores the role of a super-isentropic melting curve in the deep mantle (Section S2.1 in the supplementary information, mid_mantle_video.mp4).

- Figure 1 : Temperature and FeO fields.
- Figure 2 : Trace Elements field.
- Super isentropic melting (Section S2.1) : Temperature and FeO fields.

References

[1] Nabiei, F. *et al.* Investigating magma ocean solidification on earth through laser-heated diamond anvil cell experiments. *Geophysical Research Letters* e2021GL092446 (2021) .

[2] Boukaré, C.-E., Ricard, Y. & Fiquet, G. Thermodynamics of the MgO-FeO-SiO_2 system up to 140 GPa: Application to the crystallization of Earth's magma ocean. *J Geophys. Res.: Solid Earth* **120** (9), 6085–6101 (2015). URL http://dx.doi.org/10.1002/2015JB011929. https://doi.org/10.1002/2015JB011929, 2015JB011929 .

[3] Wallner, H. & Schmeling, H. Numerical models of mantle lithosphere weakening, erosion and delamination induced by melt extraction and emplacement. *International Journal of Earth Sciences* **105** (6), 1741–1760 (2016) .

[4] Ricard, Y., Bercovici, D. & Schubert, G. A two-phase model for compaction and damage: 2. applications to compaction, deformation, and the role of interfacial surface tension. *Journal of Geophysical Research: Solid Earth* **106** (B5), 8907–8924 (2001) .

[5] Oliveira, B., Afonso, J. C., Zlotnik, S. & Diez, P. Numerical modelling of multiphase multicomponent reactive transport in the earth's interior. *Geophysical Journal International* **212** (1), 345–388 (2018) .

[6] Keller, T. & Suckale, J. A continuum model of multi-phase reactive transport in igneous systems. *Geophysical Journal International* **219** (1), 185–222 (2019) .

[7] Corgne, A., Liebske, C., Wood, B. J., Rubie, D. C. & Frost, D. J. Silicate perovskite-melt partitioning of trace elements and geochemical signature of a deep perovskitic reservoir. *Geochimica et Cosmochimica Acta* **69** (2), 485–496 (2005) .

[8] Kennedy, A., Lofgren, G. & Wasserburg, G. An experimental study of trace element partitioning between olivine, orthopyroxene and melt in chondrules: equilibrium values and kinetic effects. *Earth and Planetary Science Letters* **115** (1-4), 177–195 (1993) .

[9] Kohlstedt, D. L., Bai, Q., Wang, Z.-C. & Mei, S. Rheology of partially molten rocks. *Physics and chemistry of partially molten rocks* 3–28 (2000) .

[10] Costa, A. Viscosity of high crystal content melts: dependence on solid fraction. *Geophys. Res. Lett.* **32** (22) (2005) .

[11] Jaupart, C. & Mareschal, J.-C. Heat generation and transport in the earth. *Cambridge University Press* **2**, 345–365 (2010) .

[12] Solomatov, V. S. & Moresi, L.-N. Scaling of time-dependent stagnant lid convection: Application to small-scale convection on earth and other terrestrial planets. *Journal of Geophysical Research: Solid Earth* **105** (B9), 21795–21817 (2000) .

[13] Boukaré, C.-E. & Ricard, Y. Modeling phase separation and phase change for magma ocean solidification dynamics. *Geochemistry, Geophysics, Geosystems* **18** (9), 3385–3404 (2017) .

[14] Mckenzie, D. The generation and compaction of partially molten rock. *Journal of Petrology* **25** (3), 713–765 (1984) .

[15] Abe, Y. & Matsui, T. Early evolution of the Earth: Accretion, atmosphere formation, and thermal history. *J. Geophys. Res.: Solid Earth* **91** (B13), E291–E302 (1986). URL http://dx.doi.org/10.1029/JB091iB13p0E291. https://doi.org/10.1029/JB091iB13p0E291 .

[16] Zahnle, K. J., Kasting, J. F. & Pollack, J. B. Evolution of a steam atmosphere during earth's accretion. *Icarus* **74** (1), 62–97 (1988) .

[17] Hamano, K., Abe, Y. & Genda, H. Emergence of two types of terrestrial planet on solidification of magma ocean. *Nature* **497** (7451), 607–610 (2013) .

[18] Lebrun, T. *et al.* Thermal evolution of an early magma ocean in interaction with the atmosphere. *Journal of Geophysical Research: Planets* **118** (6), 1155–1176 (2013) .

[19] Labrosse, S., Hernlund, J. & Coltice, N. A crystallizing dense magma ocean at the base of the Earth's mantle. *Nature* **450** (7171), 866–869 (2007). URL http://www.nature.com/nature/journal/v450/n7171/full/nature06355.html. https://doi.org/10.1038/nature06355 .

[20] Bower, D. J., Sanan, P. & Wolf, A. S. Numerical solution of a non-linear conservation law applicable to the interior dynamics of partially molten planets. *Physics of the earth and planetary interiors* **274**, 49–62 (2018) .

[21] Monteux, J., Andrault, D. & Samuel, H. On the cooling of a deep terrestrial magma ocean. *Earth Planet. Sci. Lett.* **448**, 140 – 149 (2016). URL http://www.sciencedirect.com/science/article/pii/S0012821X16302199. https://doi.org/http://dx.doi.org/10.1016/j.epsl.2016.05.010 .

[22] Warren, P. H. The magma ocean concept and lunar evolution. *Annu. Rev. Earth Planet. Sci.* **13** (1), 201–240 (1985) .

[23] Suckale, J., Elkins-Tanton, L. T. & Sethian, J. A. Crystals stirred up: 2. numerical insights into the formation of the earliest crust on the moon. *Journal of Geophysical Research: Planets* **117** (E8), n/a–n/a (2012). URL http://dx.doi.org/10.1029/2012JE004067. https://doi.org/10.1029/2012JE004067, e08005 .

[24] Martin, D. & Nokes, R. Crystal settling in a vigorously converting magma chamber. *Nature* **332** (6164), 534–536 (1988) .

[25] Martin, D. & Nokes, R. A fluid-dynamical study of crystal settling in convecting magmas. *Journal of Petrology* **30** (6), 1471–1500 (1989) .

[26] Tonks, W. & Melosh, H. The physics of crystal settling and suspension in a turbulent magma ocean. *Origin of the Earth* **1**, 151–174 (1990) .

[27] Solomatov, V. S. & Stevenson, D. J. Suspension in convective layers and style of differentiation of a terrestrial magma ocean. *J. Geophys. Res: Planets* **98** (E3), 5375–5390 (1993). URL http://dx.doi.org/10.1029/92JE02948. https://doi.org/10.1029/92JE02948 .

[28] Solomatov, V. S. & Stevenson, D. J. Nonfractional crystallization of a terrestrial magma ocean. *J. Geophys. Res: Planets* **98** (E3), 5391–5406 (1993). URL http://dx.doi.org/10.1029/92JE02579. https://doi.org/10.1029/92JE02579 .

[29] Verhoeven, J. & Schmalzl, J. A numerical method for investigating crystal settling in convecting magma chambers. *Geochemistry Geophysics Geosystems* **10** (2009). https://doi.org/{10.1029/2009GC002509} .

[30] Le Bars, M., Wieczorek, M. A., Karatekin, Ö., Cébron, D. & Laneuville, M. An impact-driven dynamo for the early moon. *Nature* **479** (7372), 215–218 (2011) .

[31] Maas, C. & Hansen, U. Effects of earth's rotation on the early differentiation of a terrestrial magma ocean. *Journal of Geophysical Research: Solid Earth* **120** (11), 7508–7525 (2015). URL http:https://dx.doi.org/10.1002/2015JB012053. https://doi.org/10.1002/2015JB012053 .

[32] Maas, C. & Hansen, U. Dynamics of a terrestrial magma ocean under planetary rotation: A study in spherical geometry. *Earth and Planetary Science Letters* **513**, 81–94 (2019) .

[33] Huppert, H. E. & Sparks, R. S. J. The fluid dynamics of a basaltic magma chamber replenished by influx of hot, dense ultrabasic magma.

Contributions to Mineralogy and Petrology **75** (3), 279–289 (1981) .

[34] Lavorel, G. & Le Bars, M. Sedimentation of particles in a vigorously convecting fluid. *Physical Review E* **80** (4), 046324 (2009) .

[35] Pandey, A., Verma, M. K. & Mishra, P. K. Scaling of heat flux and energy spectrum for very large prandtl number convection. *Physical Review E* **89** (2), 023006 (2014) .

[36] Dygert, N., Lin, J.-F., Marshall, E. W., Kono, Y. & Gardner, J. E. A low viscosity lunar magma ocean forms a stratified anorthitic flotation crust with mafic poor and rich units. *Geophysical Research Letters* **44** (22), 11,282–11,291 (2017). URL https://agupubs.onlinelibrary.wiley.com/doi/abs/10.1002/2017GL075703. https://doi.org/https://doi.org/10.1002/2017GL075703, https://agupubs.onlinelibrary.wiley.com/doi/pdf/10.1002/2017GL075703 .

[37] Solomatov, V. S. & Stevenson, D. J. Kinetics of crystal growth in a terrestrial magma ocean. *J. Geophys. Res: Planets* **98** (E3), 5407–5418 (1993). URL http://dx.doi.org/10.1029/92JE02839. https://doi.org/10.1029/92JE02839 .

[38] Elkins-Tanton, L. T. Magma oceans in the inner solar system. *Annu. Rev. Earth Planet. Sci.* **40**, 113–139 (2012). https://doi.org/{10.1146/annurev-earth-042711-105503} .

[39] Mcdonough, W. & Sun, S. The composition of the earth. *Chemical Geology* **120** (3-4), 223–253 (1995). https://doi.org/10.1016/0009-2541(94)00140-4 .

[40] Bédard, J. Partitioning coefficients between olivine and silicate melts. *Lithos* **83** (3-4), 394–419 (2005) .

[41] Katz, R. F. & Rudge, J. F. The energetics of melting fertile heterogeneities within the depleted mantle. *Geochemistry, Geophysics, Geosystems* **12** (10) (2011) .

[42] Fiquet, G. *et al.* Melting of peridotite to 140 gigapascals. *Science* **329** (5998), 1516–1518 (2010). URL http://www.sciencemag.org/content/329/5998/1516.abstract. https://doi.org/10.1126/science.1192448, http://www.sciencemag.org/content/329/5998/1516.full.pdf .

[43] Salvador, A. & Samuel, H. Convective outgassing efficiency in planetary magma oceans: insights from computational fluid dynamics. *Icarus* **390**, 115265 (2023) .

[44] Parai, R. A dry ancient plume mantle from noble gas isotopes. *Proceedings of the National Academy of Sciences of the United States of America* **119** (29), 1–9 (2022). https://doi.org/10.1073/pnas.2201815119 .
